# Methamphetamine Use and Suicide: A Case Report and Brief Review of Literature

**DOI:** 10.7759/cureus.64835

**Published:** 2024-07-18

**Authors:** Eduardo D Espiridion, Lily Charron

**Affiliations:** 1 Psychiatry, Drexel University College of Medicine, Philadelphia, USA; 2 Psychiatry, Reading Hospital, West Reading, USA

**Keywords:** intoxication, public health and safety, drug addiction, suicide and depression, methamphetamine use

## Abstract

Recreational use of methamphetamines has greatly increased in frequency across the world. Like other stimulants, methamphetamines can cause several health consequences, and their addictive nature can lead to psychiatric disorders. Suicidal ideation and attempts are common in methamphetamine users and have become a leading cause of death next to incidental overdoses. In the current report, we review a case of a methamphetamine user who attempted suicide by jumping off a bridge. Further, we contextualize this case by reviewing recent literature on the relationship between methamphetamine use and suicide.

## Introduction

Methamphetamine (MA) use has increasingly become a global issue with a growing frequency of users and health complications [1,2]. Second only to global marijuana use, MA can have detrimental health consequences. Like many other drugs of abuse, MA overdose can be fatal, and in fact, many MA-related deaths are from overdose. This fact may be complicated by the frequency at which MA users abuse other substances, most commonly marijuana, alcohol, and opiates [3].

Mental and behavioral issues such as anxiety, depression, suicidal ideation, and psychosis are common among MA users [3,4]. The frequency of these disorders means rates of suicide are higher than patient populations with no psychiatric issues. One study found injection MA use increased the chances of a suicide attempt by 80% compared to non-MA injection users [5]. Another analysis of completed MA-related suicides found that a quarter of cases had previous suicide attempts [6]. As the number of MA users has increased, so have the cases of MA-related suicide. In the United States, the number of hospital admissions for suicidal ideation of patients with MA dependence has increased 16-fold from 2008 to 2019 [7].

MA use is more common among men than women [2]. Other risk factors include comorbidities such as HIV/AIDS, criminal justice involvement, and lower annual household income [2]. While men are more likely to use MA, within the population of MA users, young women (<25 years) with psychiatric comorbidities were most at risk of suicide [8]. Women are also more likely to seek medical attention for suicidal ideation [9]. MA users with bipolar disorder have the highest suicide risk, followed by users with depression, schizophrenia, and anxiety disorders [8].

In the present study, we describe a case of a young man with a history of MA use disorder who attempted suicide by jumping off a bridge. The case analysis serves as a conduit to review the existing literature on suicide risk and completion by people with MA use disorder.

## Case presentation

**Figure 1:**
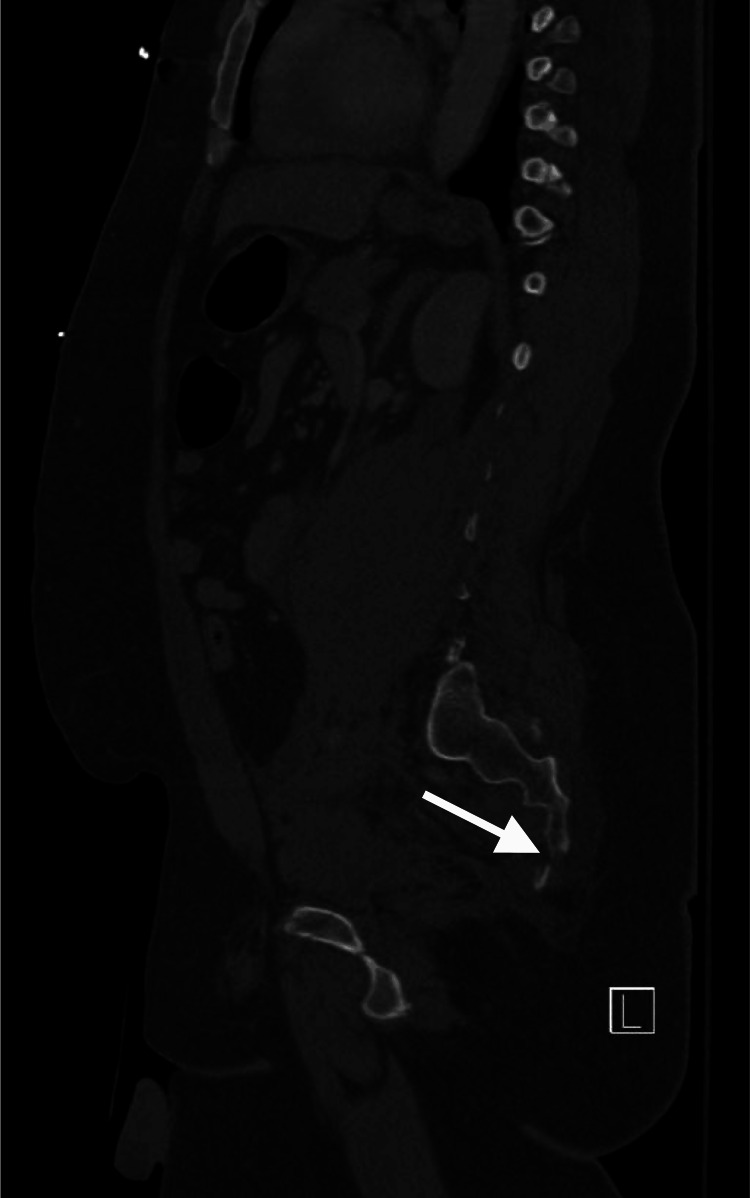
CT scan of the pelvis shows a distal sacral fracture.

**Figure 2:**
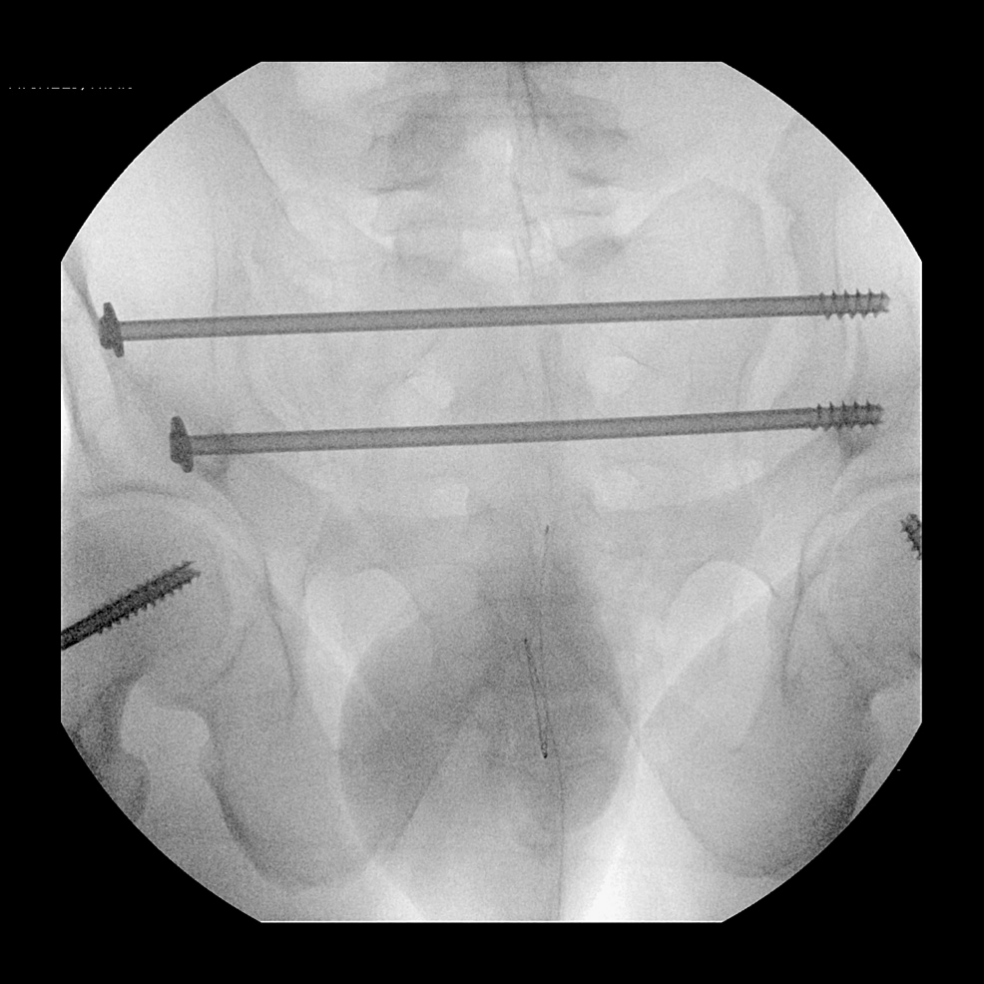
CT scan of the pelvis shows an ORIF of the fracture through the sacroiliac joint with postoperative anatomic alignment. ORIF: open reduction internal fixation

**Figure 3:**
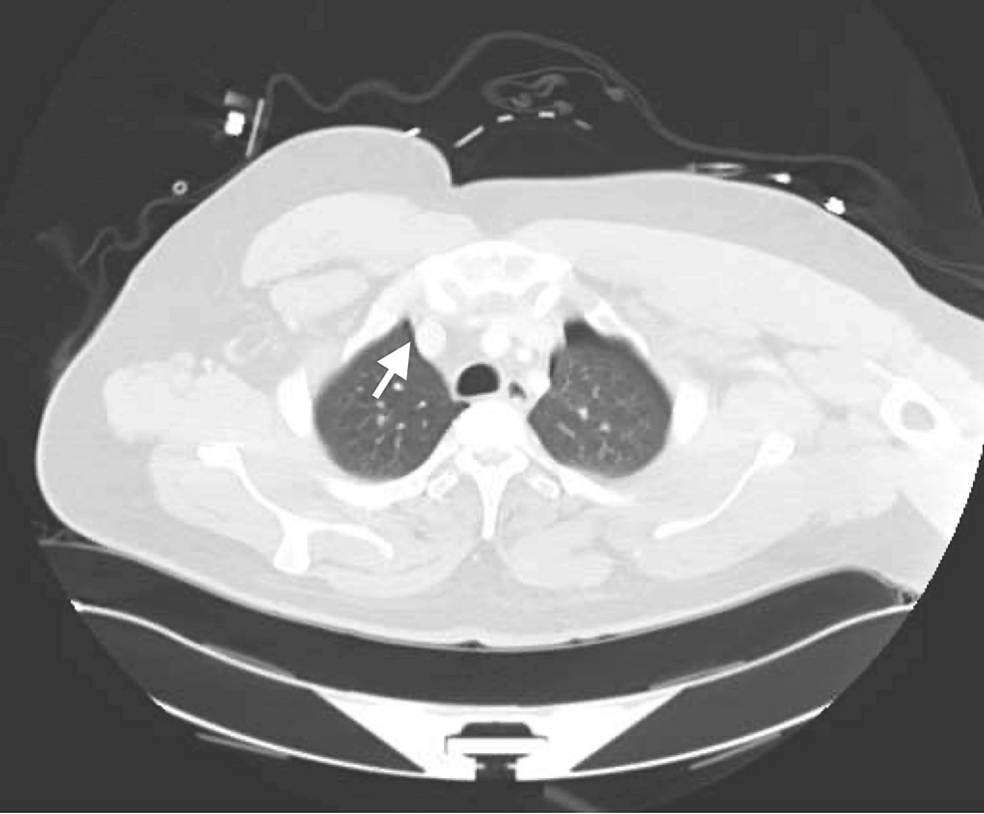
CT scan of the abdomen shows a left retroperitoneal hemorrhage.

**Figure 4:**
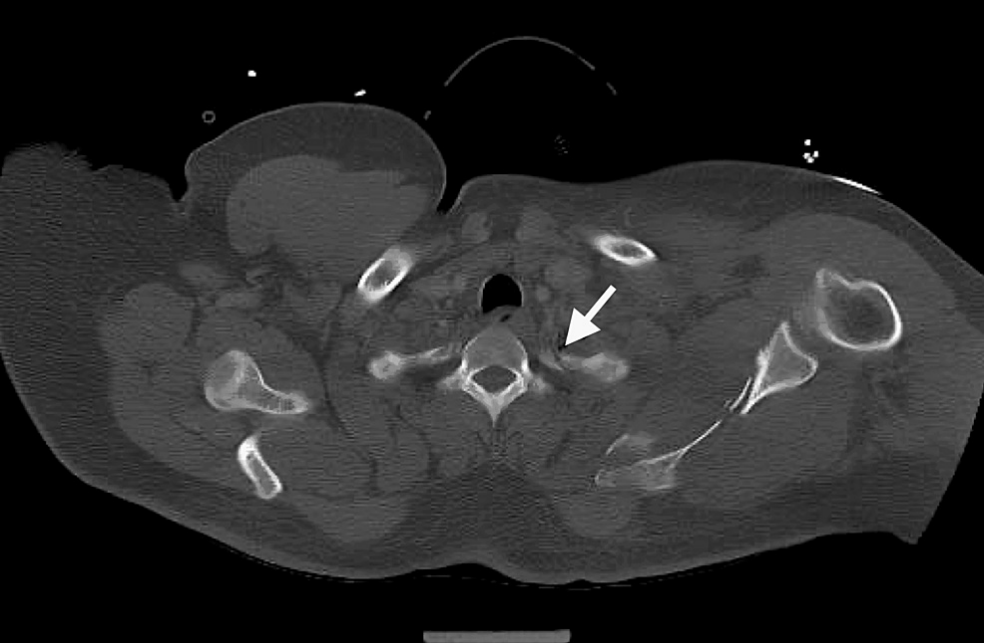
CT scan of the abdomen shows fracture of transverse process of lumbar spine.

A 30-year-old single unemployed male presented to the emergency department (ED) of a local hospital after jumping off a 30-foot bridge. He was conscious after the fall. The fall resulted in fractures in his sacral vertebrae, pubic rami, and several ribs (Figure 1). There was an improved postoperative anatomic alignment of the sacroiliac joint after an open reduction and internal fixation surgery (Figure 2). The fall also resulted in traumatic retroperitoneal hematoma (Figure 3). There were fractures of the transverse and spinous processes of his lumbar vertebra as well (Figure 4).

After being medically stabilized, the patient reported that the jump was a planned suicide attempt. He claimed he was severely depressed due to his inability to control his MA use and his conflict with his family. He admitted having a sad mood, mood lability, irritability, poor impulse control, sleeping disturbances, anhedonia, isolative behaviors, social withdrawal, and active suicidal ideations. The patient admitted to eating “ice” prior to jumping off the bridge. When questioned, the patient was able to give his first name but claimed he did not remember his last name. Even after the hospital staff were able to identify the patient’s full name, he denied that that was his name. The patient claimed he was “hyped up” and appeared paranoid and internally preoccupied. He denied any hallucinations. He was restless but was not aggressive. 

The patient insisted that he had no significant medical issues; however, his admission laboratory work-up showed leukocytosis and hyperglycemia and a positive toxicology screen for amphetamines (Tables 1, 2). He admitted to a history of several inpatient psychiatric admissions. He had been diagnosed with depressive disorder and MA use disorder during those admissions. He had recently left an inpatient facility a few days before he jumped off the bridge. He had a past history of several suicide attempts by cutting his arm several times as well as an attempted hanging. The patient had no current medical or psychiatric provider. He was noncompliant with psychiatric medications. He was lost to psychiatric follow-up appointments since he was discharged from the psychiatric hospital. He reported a long history of MA use. He was on Disability and claimed he lived with his sibling. The patient was administered several medications for pain management, including acetaminophen, lidocaine, methocarbamol, and oxycodone.

**Table 1:** CBC with differential and basic metabolic panel WBC: white blood cells; RBC: red blood cells; IMM GRAN: immature granulocytes; CBC: complete blood count

Component	Value	Reference Range
WBC	29.2x10^3^/uL	4.8-10.8x10^3^/uL
RBC	4.44x10^6^/uL	4.50-6.10x10^6^/uL
Hemoglobin	13.9 g/dL	14.0-17.5 g/dL
Hematocrit	41.8%	39.0-53.0%
Platelets	287x10^3^/uL	130-400x10^3^/uL
Neutrophils	26.05x10^3^/uL	2.00-8.00x10^3^/uL
Lymphocytes	1.48x10^3^/uL	0.70-5.20x10^3^/uL
Monocytes	1.18x10^3^/uL	0.10-1.30x10^3^/uL
Eosinophils	0.01x10^3^/uL	0.04-0.54x10^3^/uL
Basophils	0.06x10^3^/uL	0.00-0.21x10^3^/uL
IMM GRAN	0.42x10^3^/uL	0.00-0.03x10^3^/uL
Glucose	198 mg/dL	74-99 mg/dL

**Table 2:** Urinalysis and urine toxicology screen THC: tetrahydrocannabinol

Component	Value	Reference Range
Amphetamine	Positive	Negative
THC	Positive	Negative
Glucose	100 mg/dL	Negative
Bilirubin	Small	Negative
Ketones	Trace	Negative
Protein	300 mg/dL	Negative
Leukocytes	Trace	Negative

On the recommendation of the psychiatrist, the patient was started on lorazepam and olanzapine as needed. After a few days of monitoring, the patient was calmer and more cooperative. He did not remember his initial meeting with the psychiatrist. He maintained he had wanted to die when he jumped off the bridge. The patient was consistently monitored on a one-to-one basis until he was medically cleared and subsequently admitted into an inpatient psychiatric facility. The patient acknowledged that whenever he was under the influence of MA, he became extremely paranoid and guarded. Before he was transferred to an inpatient psychiatric unit, the patient agreed with the psychiatric treatment plan after his insight improved and he realized his lack of a social support system. He eventually agreed to start referrals to mental health and addiction services in the community. 

## Discussion

The present case report of a MA user attempting suicide illustrates just one data point in the epidemic of MA use disorder and suicide. We review the recent literature on MA use and suicide to better understand those who are at risk and initiate effective preventative measures.

Several studies on the comorbidities and causes of death of MA users were performed in Taiwan using the National Health Insurance Research Data (NHIRD). The NHIRD covers medical information for 99% of the population in Taiwan and is linked to the Taiwan Death Registry (TDR). One study found that from a MA-using population of 21,809, 47.8% had at least one psychiatric disorder, with older women being the demographic most likely to have a psychiatric disorder [8]. As previously summarized, young women are the primary group of MA users most at risk for suicidal ideation and attempt and are most likely to seek medical attention [8,9]. The most common comorbid psychiatric disorders observed were depression, anxiety, bipolar disorder, and schizophrenia. The 21,809 patients were followed for a 17-year period. After follow-up, investigators found that users with at least one psychiatric disorder had a higher rate of all-cause mortality and were more likely to die of unnatural causes than natural causes. Of the deaths by unnatural causes, over half were suicides, and patients with any psychiatric disorder were 2.27 times more likely to die by suicide. These results demonstrate the pervasiveness of suicide in MA users, especially young women, and the need to target this population for prevention.

The same database was used to further investigate all-cause and suicide mortality in this population [10]. Of all causes of death in MA users, suicide had the highest standardized mortality rate (SMR), followed by neurological diseases. They observed that cumulative completed suicides were higher for male users than female users. This may be due to the finding that men were at higher risk for multiple substance use, which had the highest SMR for all-cause mortality. Like previous results, women were found more likely to have non-substance use psychiatric disorders. For both genders, suicide attempts were most frequent during the first year after diagnosis of MA use disorder.

A study in Iraq further delved into the risk factors for suicide ideation among MA users [11]. It is important to note that the study focused on users of crystal MA, which is the purified solid form of the drug that is typically smoked or snorted. Unlike the previously mentioned studies, this analysis found no significant difference in suicidal ideation between men and women or between age groups, though this result may be due to a scarcity of women within the sample. The researchers found that individuals are at higher risk for suicide if they are chronic users (>1 year of use), suffer from visual hallucinations, have episodes of aggression, and abuse other illicit substances except for alcohol. Interestingly, the report found an inverse relationship between suicide risk and past trauma in MA users. The study also found an increased suicide risk for users with family members who were also drug users, and those who used crystal MA in social settings.

MA users adopt several methods of suicide that may provide a target to prevent MA-related suicides. Though many suspect MA-related suicides to be done in a state of psychosis, the majority of MA-related suicides are due to intentional overdoses of MA [10]. Other methods of suicide more common among MA users than the general population include charcoal burning, hanging, drowning, firearms, cutting, and jumping from a high place. The most common method found to be used by female MA users was jumping from high places, while the most common methods employed by male users were hanging and charcoal burning. Though roughly half of the suicides in the Taiwanese study by Lee et al. were nonviolent [10], this distribution differs from other countries. A report of postmortem MA-related deaths in Australia found that almost 70% of the suicides were by hanging [6]. Other violent methods used included exsanguination and gunshots, and all these methods exceeded the frequency of their use in the general population. In the study, toxicology did not find any significant differences in MA levels by the method of suicide, though prescription medications were commonly found including sedatives and antidepressants. Violence is nothing new to the public perception of MA users, though few studies refute that there is a connection between stimulant drug use and violent behavior [12].

The methods by which MA is used may also have an impact on suicide risk. An open prospective cohort known as the Vancouver Injection Drug Users Study (VIDUS) comparing suicide attempts and completions between intravenous (IV) and non-IV users found that those who had a history of injecting MA via IV were more likely to attempt or complete suicide than non-IV MA users [13]. Those who were younger and had been injecting for a shorter amount of time were more likely to attempt suicide in this sample. Interestingly, polydrug use neither increased nor decreased suicide risk among MA users. Finally, the researcher, Hypse, examined whether there was a dose-dependent effect of injection on suicide. They found that infrequent injection and frequent injection had significantly higher rates of suicide compared to those who had periods of no MA injection. Importantly, there are many underlying factors such as IV users being more likely to have a suicide attempt and ideation prior to any MA use than non-IV users that may contribute to this discrepancy [13]. The results indicate that this is an area of MA and suicide research that must be further explored.

Studies of the utilization of psychiatric and medical care found an increase in healthcare visits by MA users in the few months prior to their suicide. A case-control study found that 87% of MA users who committed suicide used health services in the three-month period prior to their death [14]. This statistic was higher than the healthcare use of other substance abuse-related suicides and may reflect a stronger desire for MA users to seek help and prevent self-harm. The visits prior to their suicides were most frequently to the emergency and psychiatric departments, indicating targets for future preventative measures. Interestingly, this study by Lee et al. also identified several physical and psychiatric comorbidities present in the months prior to MA-related suicides [14]. The researchers found pneumonia to be one of the more common acute disorders, illustrating a decline in health shortly before suicide. It is also important to note that these statistics may be an underestimation as many MA users likely avoid seeking healthcare to avoid legal consequences or social stigma.

There are several social and medical risks and protective factors for suicide in MA users. A nested case-control study found that those who were financially independent were at lower risk for suicide [15]. Visual hallucinations increase the risk for suicide. Unsurprisingly, previous suicide attempts and depression are both risk factors for MA-related suicide. Factors that initiate using MA in the first place include stressful life events, psychological trauma, and peer pressure, as well as family problems, financial hardship, and marital conflicts [11]. Understanding these risk factors and triggers will improve screening and treatment for suicide in MA users.

Table 3 shows the review of the recent literature on MA use and suicide to better understand people who are at risk and how to initiate effective preventative measures.

**Table 3:** Summary of the comparative analysis of the reviewed literature MA: methamphetamine; SMR: standardized mortality rate

Authors	Title	Cohort (Country)	Findings
Darke et al., 2019 [6]	Completed suicide among methamphetamine users: a national study.	Postmortem MA deaths (Australia)	Almost 70% of MA-related suicides were hangings; other suicide methods included exsanguination and gunshots.
Fang et al., 2023 [8]	Influence of baseline psychiatric disorders on mortality and suicide and their associations with gender and age in patients with methamphetamine use disorder.	MA-using population from the Taiwan National Health Insurance Research Data (NHIRD); follow-up over a 17-year period (Taiwan)	47.8% had at least one psychiatric disorder; those with at least one psychiatric disorder had higher all-cause mortality and more likely to die of unnatural causes, and more likely to die by suicide.
Lee et al., 2021 [10]	All-cause and suicide mortality among people with methamphetamine use disorder: a nationwide cohort study in Taiwan.	MA-using population from the Taiwan National Health Insurance Research Data (NHIRD) (Taiwan)	Suicide had the highest SMR; completed suicides were higher for men than women; men were at higher risk for polysubstance use disorder, which had the highest SMR for all-cause mortality; women were more likely to have non-substance use psychiatric disorders; suicide attempts were most common during the first year after MA use disorder. MA–related suicides were most often intentional overdoses, followed by other violent methods.
Al-Imam et al., 2023 [11]	Risk factors of suicidal ideation in Iraqi crystal methamphetamine users.	Crystal MA users from Baghdad Teaching Hospital and Ibn-Rushd Teaching Hospital (Iraq)	Users were at higher risk for suicide when they were chronic users (>1 year of use), suffer from visual hallucinations, have episodes of aggression, and abuse other substances except alcohol; there was an inverse relationship between suicide risk and past trauma; there was an increased suicide risk in users with family members who also used and those who used socially. Factors that initiated first MA use include stressful life events, psychological trauma, peer pressure, family problems, financial hardship, and marital conflicts.
Hypse, 2018 [13]	Suicide rates between methamphetamine users who inject versus non-injectors.	Open prospective cohort - Vancouver Injection Drug Users Study (VIDUS) (Canada)	Users who inject MA intravenously (IV) were more likely to attempt and complete suicide than non-IV users; users who were younger and injecting for a shorter period of time were at a higher suicide risk; They found a dose-dependent relationship between injection frequency and suicide risk where more frequent IV MA use indicated a higher suicide risk.
Lee et al., 2022 [14]	Healthcare utilization and psychiatric and physical comorbidities before suicide mortality in patients with methamphetamine use disorder: a nationwide case-control study.	MA-using population from the Taiwan National Health Insurance Research Data (NHIRD) (Taiwan)	87% of MA users who committed suicide used health services in the three-month period prior to their death, with visits most frequently in the emergency and psychiatry departments; there was also an increase in acute illnesses prior to suicide, most commonly pneumonia.
Kuo et al., 2010 [15]	Risk and protective factors for suicide among patients with methamphetamine dependence: a nested case-control study.	Inpatients with MA dependence in a northern Taiwanese hospital (Taiwan)	Users who were financially independent had a lower suicide risk; visual hallucinations, depression, and previous suicide attempts increased suicide risk.

There are a few limitations to our report. As this was only a brief foray into the literature on MA use and suicide, it is not a complete list of the literature published. We did not include analyses focused on adolescent users as the suicide rates for adolescents tend to be higher [4]. Further, it stands to reason that in all analyses of MA users, their use is underreported, as users may want to avoid legal consequences or social stigma.

## Conclusions

MA use has sharply increased in recent years and threatens to continue. The number of medical and psychiatric consequences of MA use make this drug a source of health crisis. Among these consequences, suicidal ideation and suicide completion are highly prevalent. With the increasing comorbidity with different issues, it is of the utmost importance to better understand these patients in order to provide quality care and preventative measures.
